# Process Design of Vinyl-Coated Metal Sheet Stamping for Prevention of Delamination and Wrinkling by DNN-Based Multi-Objective Optimization

**DOI:** 10.3390/ma18245589

**Published:** 2025-12-12

**Authors:** Min-Gi Kim, Jae-Chang Ryu, Chan-Joo Lee, Jin-Seok Jang, Dae-Cheol Ko

**Affiliations:** 1 Department of Nanomechatronics Engineering, Pusan National University, Busan 46241, Republic of Korea; kkkkk@pusan.ac.kr; 2 Industrial Liaison Innovation Center, Pusan National University, Busan 46241, Republic of Korea; 3 Advanced Mobility Components Group, Korea Institute of Industrial Technology, Daegu 42994, Republic of Korea; cjlee80@kitech.re.kr (C.-J.L.); jsjang@kitech.re.kr (J.-S.J.)

**Keywords:** vinyl-coated metal (VCM), deep neural networks (DNN), multi-objective optimization, delamination limit diagram (DLD), finite element (FE) simulation

## Abstract

The increasing use of vinyl-coated metal (VCM) sheets in home appliances requires robust forming processes to prevent defects such as delamination and wrinkling, especially under elevated temperatures and humidity. This study presents a deep neural network (DNN)-based multi-objective optimization framework to determine optimal stamping parameters for VCM sheets. A delamination limit diagram (DLD) is experimentally established by combining limit dome height tests with immersion tests, defining the critical strain boundary under environmentally conditions. A finite element (FE) based dataset of four process variables was then used to train a DNN surrogate model with high predictive accuracy. Using the trained DNN model, Pareto-based optimization identifies nondominated solutions balancing delamination and wrinkling. The optimal condition was validated by FE simulation, confirming simultaneous suppression of both defects within the DLD. The proposed DNN–Pareto framework provides and efficient and reliable tool for defect prediction and optimization in VCM stamping, ensuring high surface quality and environmental durability.

## 1. Introduction

In the home appliance industry, the demand for lightweight structures with enhanced corrosion resistance and high-quality surface finishes has led to the increased use of coated steel sheets [1]. Among these, vinyl-coated metal (VCM) sheets have been widely adopted for the exterior panels of appliances owing to their excellent surface gloss and corrosion resistance. However, these multilayer materials are prone to surface defects such as delamination and wrinkling because of the repetitive tensile and compressive stresses acting at the interface during forming [2,3]. Delamination occurs when the interfacial strength between the coating and substrate exceeds the adhesion strength or when interfacial separation is induced by thermal or mechanical stresses [4]. However, wrinkles result from local instability under compressive stresses acting on the sheet thickness. These two defects conflict with each other. A reduction in interfacial stresses can suppress delamination but may promote wrinkling, whereas an increase in restraining stress to prevent wrinkling can accelerate delamination [5,6]. Therefore, the achievement of defect-free formability in VCM stamping requires the simultaneous optimization of both delamination and wrinkling behaviors, leading to multi-objective optimization.

Furthermore, when VCM products are exported, they are exposed to high-temperature and high-humidity environments, particularly during trans-equatorial shipments. These environmental conditions significantly affect the interfacial adhesion of the coating. Elevated temperatures reduce the elastic modulus of the vinyl layer, whereas increased humidity causes interfacial swelling, both of which degrade adhesion and increase the possibility of delamination [7,8]. Thus, formability evaluations performed only under room-temperature conditions are insufficient to guarantee product reliability in actual service environments. To address this limitation, the delamination behavior of VCM sheets must be evaluated under realistic environmental conditions. However, the experimental evaluations of all possible combinations of process parameters are time-consuming and costly. Therefore, an integrated framework that combines finite-element (FE) simulation-based data with an efficient surrogate modeling approach is required to enable the rapid predictive analysis and optimization of complex forming behaviors.

While mechanistic approaches based on fracture mechanics such as cohesive zone models, energy release rate criteria, and analytical wrinkle theories offer fundamental insights into interfacial failure, their direct application to multilayer VCM systems presents significant challenges. Accurate calibration of these models requires layer-specific micro-parameters that are rarely available for commercial sheets, and the explicit modeling of interfaces drastically increases computational costs, rendering iterative optimization practically intractable. To address these constraints, this study adopts a phenomenological engineering approach. Similar to the FLD widely accepted in sheet metal forming, the strain-based delamination limit diagram (DLD) and wrinkle limit diagram (WLD) are employed as tractable frameworks. These diagrams serve as empirical failure envelopes that implicitly encapsulate the complex material response and environmental degradation within measurable strain boundaries.

Accordingly, this study aims to achieve two primary objectives. First, DLD is established to quantify the critical strain conditions that induce coating and substrate separation under environmental conditions [9]. Second, the reliability of the established DLD is experimentally verified through forming tests, and the results are used to determine the optimal process conditions that simultaneously minimize delamination and wrinkling defects. For this purpose, a deep neural network (DNN)-based multi-objective optimization framework is employed using an FE simulation dataset. The DNN model, which effectively captures the complex nonlinear relationships between process parameters and defect responses, is constructed by defining process variables as inputs and using delamination and wrinkling as output objectives [10,11]. Based on the trained DNN model, Pareto-based multi-objective optimization is performed to identify the optimal forming conditions that minimize both defects (delamination and wrinkling) under environmentally relevant conditions.

The overall objective of this research is to establish and validate a DLD and develop a DNN-based multi-objective optimization framework to predict and optimize forming defects in VCM stamping. Using this integrated approach, stable formability and superior surface quality can be achieved even under the environmental conditions encountered during production and transportation, thereby providing a robust technological foundation for the design and manufacture of VCM products.

## 2. Materials and Methods

### 2.1. Mechanical Properties of the VCM Sheet

**Figure 1:**
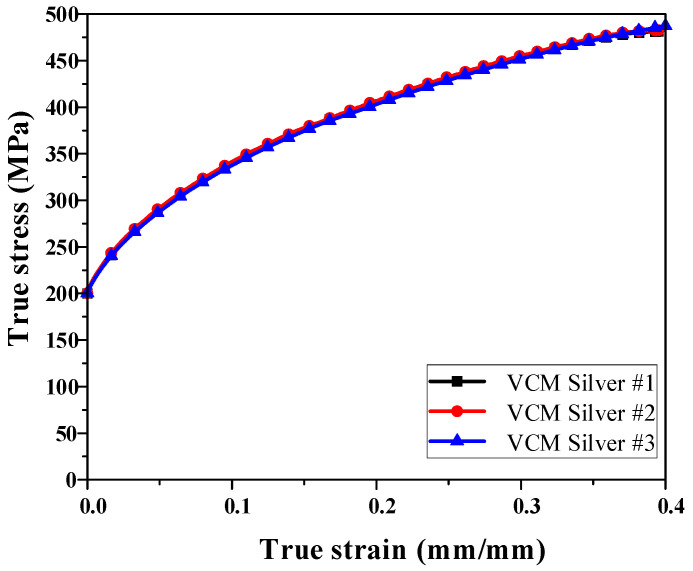
Results of uniaxial tensile test for VCM sheet.

Accurate mechanical properties of the applied material must be obtained to perform FE simulations of the stamping process using VCM sheets (BN Steela, Busan, Republic of Korea). A commercial VCM sheet with a thickness of 0.8 mm was used. The mechanical properties of the VCM sheets were evaluated via tensile tests at room temperature. The tensile specimens were fabricated by laser cutting, following the specifications of the ASTM E8 standard [12]. Tensile tests were conducted using a universal testing machine (MTS Systems, Eden Prairie, MN, USA) with a capacity of 10 t. A crosshead speed of 2 mm/min was applied during the test to ensure quasi-static loading. The resulting strain–stress curves exhibited a consistent trend among the specimens, as illustrated in Figure 1. The average mechanical properties obtained from the tests, such as yield strength, ultimate tensile strength, and elongation, were used as input data for the FE simulations. These properties were implemented in the stamping analysis to accurately represent the deformation behavior of the VCM sheet under the forming conditions.

### 2.2. Establishment of Delamination Limit Diagram

**Figure 2:**
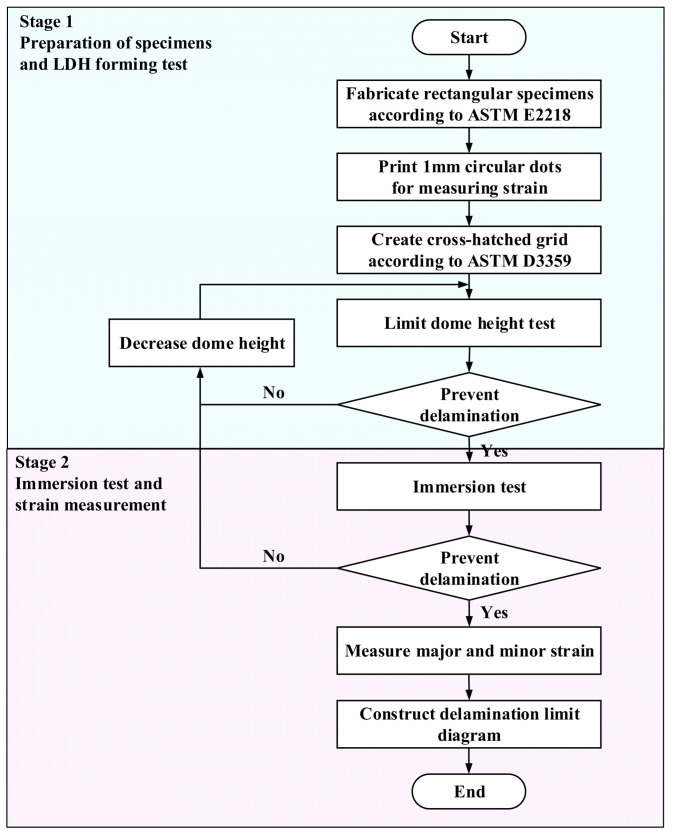
Procedure for establishing DLD through LDH test and immersion test [13,14].

To quantitatively evaluate the delamination behavior of the VCM sheets during forming, a DLD was established to represent the critical conditions for delamination under various deformation modes. The DLD is conceptually similar to the conventional FLD, in which combinations of major and minor strains define the threshold for failure. However, unlike the FLD, which represents fracture initiation, the DLD defines the strain limits at which interfacial delamination occurs between the coating and substrate. The overall procedure for constructing the DLD consists of two main stages: conducting limit dome height (LDH) forming tests according to ASTM E2218 [13] and performing a standardized accelerated immersion aging (temperature–humidity exposure) test to correct and validate the strain limits under severe environmental conditions. The experimental procedure is illustrated in Figure 2.

**Figure 3:**
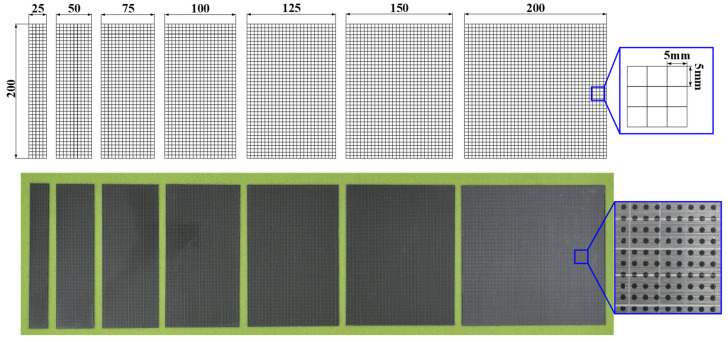
Cross-hatched specimens for constructing DLD.

First, rectangular specimens were fabricated with a fixed length of 200 mm and variable widths ranging from 25 to 200 mm, including deformation modes from uniaxial tension to biaxial tension. All specimens were machined using laser cutting, and circular dot patterns with a diameter of 1 mm were printed on the surface for the strain measurement using an optical system (ARGUS, Zeiss, Oberkochen, Germany). Because delamination is typically initiated at the edge or curvature-concentrated regions, a cross-hatched pattern was created on the coating surface to locally concentrate the interfacial stresses. The cross-hatched grid shown in Figure 3 was prepared with reference to ASTM D3359 [14] and previous studies [5,10], with a grid spacing of 5 mm × 5 mm, which enabled the clear identification of initiation and propagation during deformation.

**Figure 4:**
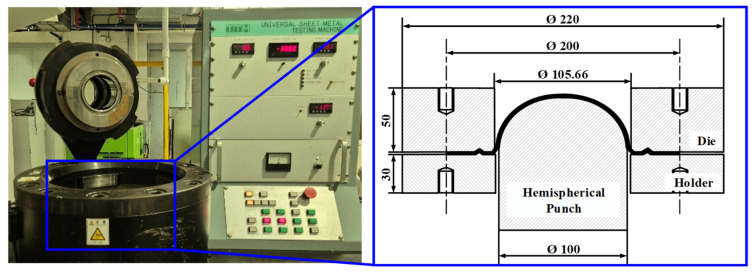
Experimental setup for limit dome height test.

Next, LDH tests were performed using the forming apparatus illustrated in Figure 4 with a punch speed of 5 mm/min and blank holding force of 200 kN. A draw bead was employed to regulate material flow. LDH tests were repeatedly conducted at various dome heights until the critical height at which delamination occurred was identified. Specifically, the dome height was first evaluated until the onset of necking and then gradually decreased in successive tests until delamination was not observed. Each specimen width was tested iteratively to ensure that the final dome height represented the maximum deformation level without delamination.

**Figure 5:**
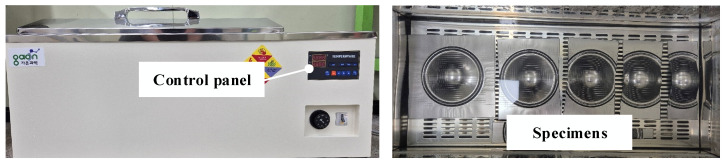
Experimental setup for immersion test.

**Figure 6:**
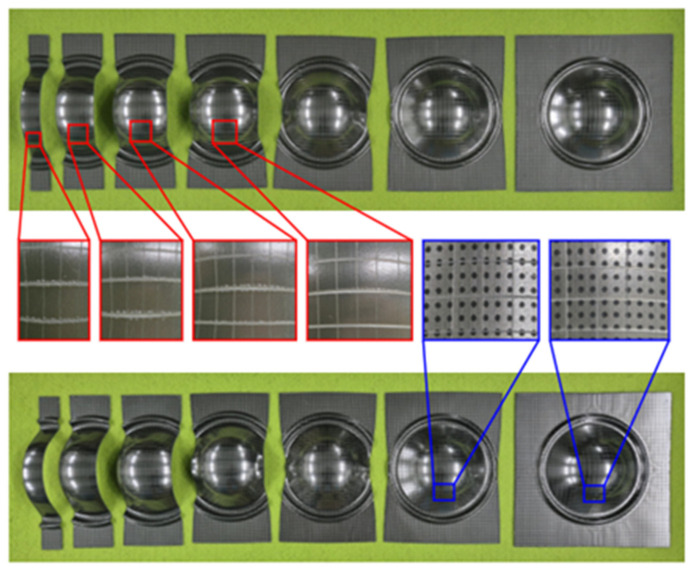
Results of specimens after LDH and immersion tests.

In the second stage, the specimens corresponding to each critical dome height were subjected to an environmental durability assessment. While service environments are typically evaluated using conventional long-duration thermal-shock cycling, such methods are impractical for the iterative testing required to construct a DLD. Therefore, a standardized accelerated aging test was employed to overcome these time constraints. This protocol, strictly following the internal qualification standards of the collaborating manufacturer, provides a severe worst-case exposure to efficiently screen adhesion degradation. As shown in Figure 5, the specimens were immersed in water at 60 °C for 30 min and then dried for 1 h. If delamination was observed after the immersion test, as shown in Figure 6, the LDH test was repeated at a lower dome height, and the immersion test was performed again. This sequential procedure was repeated for all the specimens with seven widths until delamination was not detected under the prescribed environmental conditions. The final specimens that underwent both forming and immersion tests without any observable delamination are shown in Figure 6. The specimens maintained uniform surfaces without delamination. The determined dome height represents the critical safe forming limit of a VCM sheet under harsh environmental conditions.

**Figure 7:**
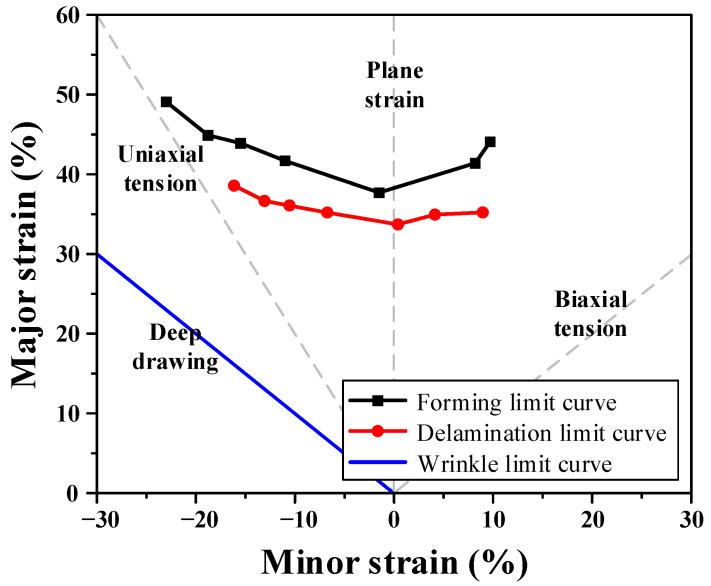
Delamination limit diagram for VCM obtained by experiments.

Finally, after confirming that no delamination occurred for each width, the deformed specimens were analyzed using an optical strain measurement system (ARGUS, Zeiss, Germany). Major and minor strains were calculated from the deformation of the printed circular grids. To ensure statistical reliability, the LDH and immersion tests were performed three times for each specimen width, and the most conservative critical strain among the repetitions was selected to define the DLD. The results were plotted with the minor strain on the *x*-axis and major strain on the *y*-axis to establish the DLD, as shown in Figure 7. The constructed DLD exhibited a shape similar to that of a conventional FLD but was located below the forming limit curve. This indicates that delamination occurred prior to fracture; therefore, the delamination limit should be considered as a critical boundary in the forming analysis of VCM sheets. Thus, the presented DLD should be interpreted as a conservative design boundary for safe forming rather than a precise, material-intrinsic limit. Consequently, the DLD must be incorporated into the stamping process design to ensure stable coating adhesion and prevent interfacial failure during forming.

Regarding compressive instability, wrinkling is a phenomenon governed primarily by the minor strain. In this study, WLD was defined based on the widely recognized wrinkle limit curve used in deep drawing mode. The deep drawing wrinkle limit curve represents the onset of out-of-plane buckling under compressive strain and has been used as an engineering criterion for predicting wrinkles in sheet metal forming. It is established through a one-to-one correspondence with the conventional deep drawing mode reported in previous studies [15,16]. By defining the wrinkle limit boundary in the principal strain space consistent with deep drawing mode, the WLD provides a conservative and robust reference for identifying wrinkle regions in stamping.

### 2.3. Deep Neural Network and Pareto-Based Multi-Objective Optimization

VCM sheets are widely used in the manufacturing of various components via stamping. However, delamination and wrinkling are the two dominant defects that occur frequently during the forming of these materials. To enhance the overall formability of a VCM sheet, both defects must be minimized simultaneously. Delamination and wrinkle are conflicting objectives; therefore, improving one typically exacerbates the other. To address this tradeoff, a multi-objective optimization framework combining a deep neural network (DNN) and Pareto optimization is proposed in this study. The proposed approach focuses on efficiently exploring the optimal solution space by comprehensively considering the complex relationships between process variables and material behavior.

**Figure 8:**
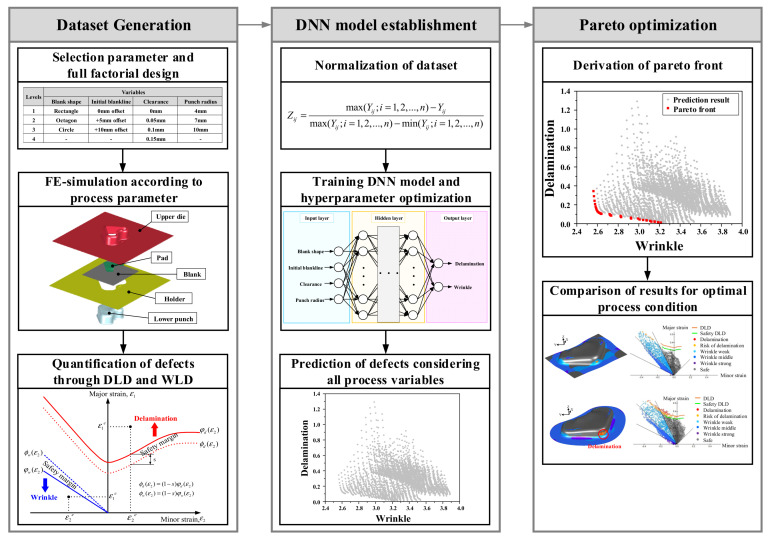
Multi-objective optimization procedure for the stamping process of VCM.

The proposed framework consists of three main stages, as illustrated in Figure 8. First, a defect dataset was constructed through FE simulation of the VCM stamping process. These simulations were systematically designed using a full factorial approach based on the number and levels of the defined process variables. In each case, the delamination and wrinkling indices were calculated based on the DLD and wrinkle limit diagram (WLD) to quantify the defect severity. Next, a DNN-based predictive model was developed to efficiently approximate the nonlinear correlations between the process parameters and defect responses. The generated dataset was normalized within the range of 0–1, and a DNN prediction model was constructed through hyperparameter optimization to ensure accurate mapping performance. The trained network served as a surrogate model that rapidly predicted delamination and wrinkling tendencies without requiring additional computationally expensive simulations. The model was used to predict delamination and wrinkling across all possible combinations of process variables within the defined design space. Finally, the Pareto front was derived using the predicted results obtained from the trained DNN model. Based on the Pareto-dominance relationships, a set of nondominated solutions representing the optimal tradeoff between delamination and wrinkling minimization was identified. The resulting Pareto front defines a feasible design space for the optimal process conditions that minimize both delamination and wrinkling. Through this procedure, the proposed DNN–Pareto optimization framework provides an effective design guideline that extends beyond the conventional single-objective formability evaluation, thereby enabling the simultaneous control and optimization of both coating delamination and wrinkling in the VCM sheet stamping process.

#### 2.3.1. Deep Neural Network

The stamping process of VCM sheets exhibits nonlinear interactions among multiple process variables. To efficiently approximate these relationships without repeatedly performing computationally expensive FE simulations, a deep neural network (DNN) was adopted as a surrogate model. The role of the DNN in this study is strictly to provide rapid prediction of delamination and wrinkling indices across the design space.

**Figure 9:**
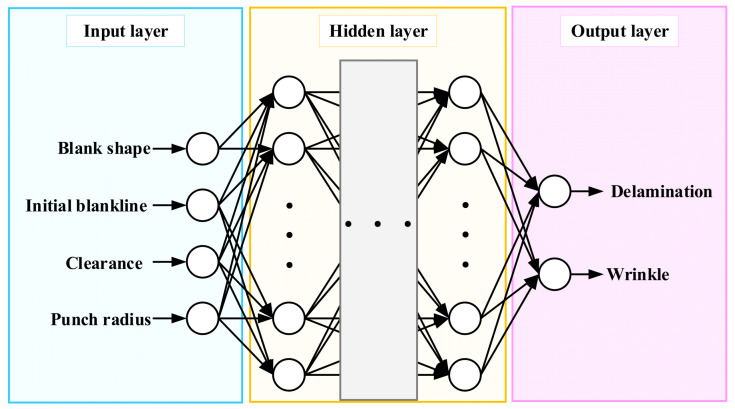
Schematic architecture of the deep neural network.

The DNN consists of an input layer, fully connected hidden layers, and two output nodes corresponding to the delamination and wrinkling, as shown in Figure 9. For each hidden layer neuron, the forward propagation is expressed as Equation (1) [17,18]:

(1) $$Y_{j}=f\left(\sum_{i=1}^{n}Y_{i}w_{ij}+b_{j}\right)$$

where *Y_i_* is the input from the previous layer, *w_ij_* is the weight, and *b_j_* is the bias. The rectified linear unit (ReLU) function was used as the activation function because of its numerical stability and suitability for nonlinear regression. The ReLU function is expressed as follows [19,20]:

(2) f(x)=max(0,x)

Training was conducted using normalized FE data, and the network parameters were optimized using Adam optimizer with early stopping to prevent overfitting [21]. The prediction accuracy of the trained DNN model was quantitatively evaluated using the mean absolute error (*MAE*), root mean square error (*RMSE*), and coefficient of determination (*R*^2^) [22]:

(3) $$MAE=\frac{1}{n}\sum \left|\hat{y}-y\right|$$

(4) $$RMSE=\sqrt{\frac{1}{n}\sum {\left(\hat{y}-y\right)}^{2}}$$

(5) $$R^{2}=1-\left(\frac{\sum {\left(y-\hat{y}\right)}^{2}}{\sum {\left(y-\bar{y}\right)}^{2}}\right),$$

where *n* is the number of data samples, *ŷ* is the predicted value, *y* is the actual value, and *ȳ* is the mean of the actual values. Once trained, the DNN replaced the FE solver within the optimization procedure, enabling rapid evaluation of design combinations while maintaining high predictive accuracy.

As a data-driven surrogate model, the DNN effectively replaced time-consuming FE simulations by rapidly predicting the objective functions. By learning the nonlinear correlations between the process variables and forming results, the model enabled the quantitative prediction of delamination and wrinkling in VCM sheet stamping.

#### 2.3.2. Pareto-Based Multi-Objective Optimization

In general optimization problems [23,24,25], the objective is to minimize or maximize a single performance function. However, in practical forming processes, multiple objectives often conflict with one another. Multi-objective optimization aims to determine an optimal set of solutions that simultaneously satisfy several conflicting objectives. In the present study, the stamping process can be formulated as a multi-objective optimization problem because both the delamination and wrinkling must be minimized, as expressed in Equation (6):

(6) $$min\;F\left(x\right)=\left[f_{1}\left(x\right),f_{2}\left(x\right)\right],\;\;x\in \Omega$$

where *f*_1_(*x*) and *f*_2_(*x*) represent the delamination and wrinkling, respectively, and Ω denotes the feasible design domain. Because these two objectives are inherently conflicting, improving one objective typically leads to the exacerbation of the other. Therefore, a single global optimum does not exist, and the Pareto optimality concept is introduced to identify the best tradeoffs among the objectives [26].

A solution is considered Pareto optimal if another solution cannot improve the objective without causing deterioration in at least one of the others. Considering two feasible solutions, $x_{a}$ and $x_{b}$, $x_{a}$ dominates $x_{b}$ if and only if [27]:

(7) $$\forall i,f_{i}\left(x_{a}\right)\leq f_{i}\left(x_{b}\right)\;and\;∋j,f_{j}\left(x_{a}\right)<f_{j}(x_{b}).$$

The set of nondominated solutions constitutes the Pareto front. Each point on the Pareto front represents a tradeoff solution that balances the competing objectives of delamination and wrinkling. Because none of these solutions dominate the other, each point can be regarded as optimal from a different design perspective. Designers can select the most suitable process conditions based on specific priorities, such as minimizing coating failure or improving surface quality.

In this study, a DNN–Pareto-based multi-objective optimization framework was employed to determine the optimal process conditions for stamping VCM sheets. The trained DNN model efficiently represents the nonlinear correlations between the process parameters and defect responses, allowing for the rapid prediction of delamination and wrinkling tendencies. The Pareto theory provides a systematic approach for analyzing the tradeoff between the two conflicting objectives. Consequently, the proposed DNN–Pareto-based approach serves as a fundamental framework for identifying the optimal forming conditions for the environmentally induced delamination and wrinkling constraints of VCM sheets.

## 3. Multi-Objective Optimization of VCM Sheet for Stamping

### 3.1. Definition of Problem for Multi-Objective Optimization

#### 3.1.1. Definition of Design Variables

In the stamping process of VCM sheets, several controllable process parameters significantly influence the forming quality [28]. If these parameters are not appropriately selected, various defects such as fractures, delamination, or wrinkles may occur during forming. Therefore, in this study, four key design variables that could be adjusted during die design or process control were selected to establish the optimal process conditions for VCM stamping. The physical meaning and effects of each variable are as follows.

**Figure 10:**
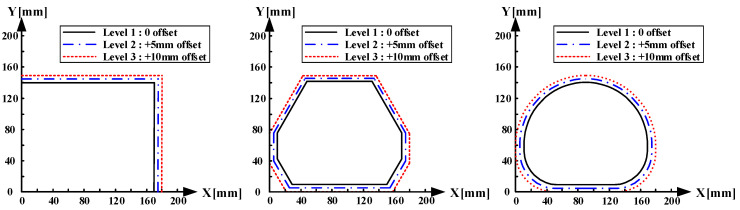
Blank shape and initial blank contour based on levels.

The first design variable was the blank shape, which directly affected the material-flow behavior and strain distribution during forming. As shown in Figure 10, three shapes were considered as the design cases. These geometries were selected by considering the trimming allowance and target product outline. The blank shape determined the strain localization and flow path of the material, which were closely related to the nonuniform deformation responsible for delamination and wrinkling. FE simulations were performed for each blank shape to analyze the influence of the geometry on the strain distribution and tendency of coating failure.

The second variable was the initial blank contour. As illustrated in Figure 10, the initial blank contour was determined based on the reference product contour and was subsequently offset by a uniform distance. This offset distance affected the material inflow and amount of tensile deformation near the sheet edge. A smaller offset may have caused material insufficiency and localized stretching, whereas an excessive offset increased the probability of wrinkling owing to excessive material inflow. In this study, three offset levels (+0, +5, and +10 mm) were considered as design variables.

**Figure 11:**
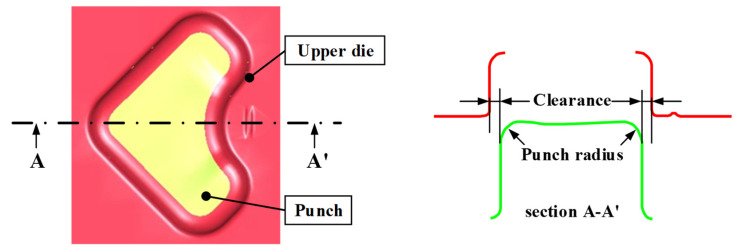
Shape of clearance and punch radius as design variables.

The third variable was the clearance between the upper die and lower punches, as shown in Figure 11. Clearance directly influenced the contact pressure, material-flow resistance, and local stress distribution. When the clearance was too small, material flow was restricted, causing excessive shear stress and delamination. Conversely, an excessively large clearance allowed for free material flow and promoted wrinkle formation. Considering the manufacturing tolerance and sheet thickness, four clearance levels (0, 0.05, 0.1, and 0.15 mm) were selected.

The final variable was the punch radius, which had a decisive influence on the curvature distribution and stress concentration during forming. As shown in Figure 11, a smaller radius intensified the local strain and interfacial stress between the coating and substrate, increasing the risk of delamination. By contrast, a larger radius allowed greater material inflow and reduced stress gradients, thereby increasing the tendency for wrinkles. Therefore, three punch radii of 4, 7, and 10 mm were selected to reflect practical die-design conditions.

These four design variables mutually affected the deformation behavior and defect formation of the VCM sheet, exhibiting a strong tradeoff relationship between delamination and wrinkling. Accordingly, a dataset was constructed by quantifying the delamination and wrinkling for all combinations of these variables, and this dataset was used for the DNN-based prediction and subsequent multi-objective optimization. The selected process variables and their levels are summarized in Table 1. A full factorial design was adopted to generate 108 simulation cases.

#### 3.1.2. Definition of Objective Functions

**Figure 12:**
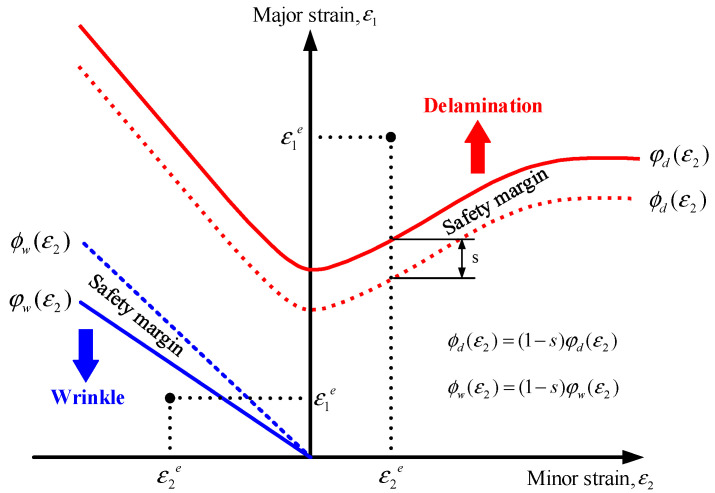
Definition of characteristic values based on the DLD and WLD.

Multi-objective optimization was conducted to simultaneously minimize the two types of defects occurring during the stamping process of the VCM sheet. To achieve this goal, two quantitative objective functions, the delamination index (*F_d_*) and wrinkle index (*F_w_*), were defined. A lower value for each index represents superior forming quality. Therefore, the optimization problem was formulated as a bi-objective minimization problem. The characteristic values of the delamination and wrinkling were obtained through FE simulations, corresponding to all combinations of the process variables described in Section 3.1.1. These indices were determined by analyzing the strain distribution of the deformed components based on the DLD and WLD. As shown in Figure 12, the DLD and WLD were plotted on the principal strain plane, where ε_1_ and ε_2_ denote the major and minor strains, respectively. The forming-limit curves corresponding to delamination and wrinkling are expressed as *ε*_1_ = $\varphi_{d}(\varepsilon_{2})$ and *ε*_1_ = $\varphi_{w}(\varepsilon_{2})$, respectively. To evaluate the process stability, safety limit curves were additionally defined to provide a conservative assessment of each defect, as follows [15,16]:

(8) $$\phi_{d}(\varepsilon_{2})=(1-s)\varphi_{d}(\varepsilon_{2})$$

(9) $$\phi_{w}(\varepsilon_{2})=(1+s)\varphi_{w}(\varepsilon_{2}),$$

where *s* denotes the safety margin determined by the designer, which was assumed to be 0.1 (10%) in this study. This safety buffer is explicitly introduced to ensure process robustness against inevitable uncertainties in manufacturing environment. For each element, when the major strain exceeded the delamination safety limit or decreased below the wrinkle safety limit, the local characteristic values *F_d_* and *F_w_* were defined as follows:

(10) $${\left\{\begin{array}{c} F_{d}^{e}=(\left|\varepsilon_{1}^{e}-\phi_{d}(\varepsilon_{2}^{e})\right|)\\ F_{d}^{e}=0 \end{array}\right.}^{p}\;\;\;\begin{array}{r} \varepsilon_{1}^{e}>\phi_{d}(\varepsilon_{2}^{e})\\ \varepsilon_{1}^{e}\leq \phi_{d}(\varepsilon_{2}^{e}) \end{array}$$

(11) $${\left\{\begin{array}{c} F_{w}^{e}=(\left|\varepsilon_{1}^{e}-\phi_{w}(\varepsilon_{2}^{e})\right|)\\ F_{w}^{e}=0 \end{array}\right.}^{p}\;\;\begin{array}{c} \varepsilon_{1}^{e}>\phi_{w}(\varepsilon_{2}^{e})\\ \varepsilon_{1}^{e}\leq \phi_{w}(\varepsilon_{2}^{e}) \end{array},$$

where *p* is an integer exponent reflecting the influence of the defect severity. The exponent was set to *p* = 2 to capture the nonlinear nature of failure escalation. Since delamination and wrinkling tend to propagate rapidly once the critical limit is exceeded, a quadratic penalty is more effective than a linear one in driving the optimization solution away from high-risk regions, consistent with widely used damage mechanics approaches [10,15]. The global characteristic values representing the overall delamination and wrinkling tendencies of the formed components were calculated using Equations (12) and (13):

(12) $$F_{d}={\left(\sum_{e=1}^{n}F_{d}^{e}\right)}^{\frac{1}{p}}$$

(13) $$F_{w}={\left(\sum_{e=1}^{n}F_{w}^{e}\right)}^{\frac{1}{p}}.$$

*F_d_* quantitatively represents the tendency for delamination, whereas *F_w_* represents the wrinkling tendency. Both indices were considered acceptable when they remained within a predefined tolerance range.

Calculated global indices were obtained for each process-parameter combination and used as target values to train the DNN prediction model. Representative examples of these calculated values are summarized in Table 2, demonstrating that the proposed quantification method effectively distinguishes between stable forming regions and potential defect zones.

#### 3.1.3. FE Simulation of the Stamping Process

**Figure 13:**
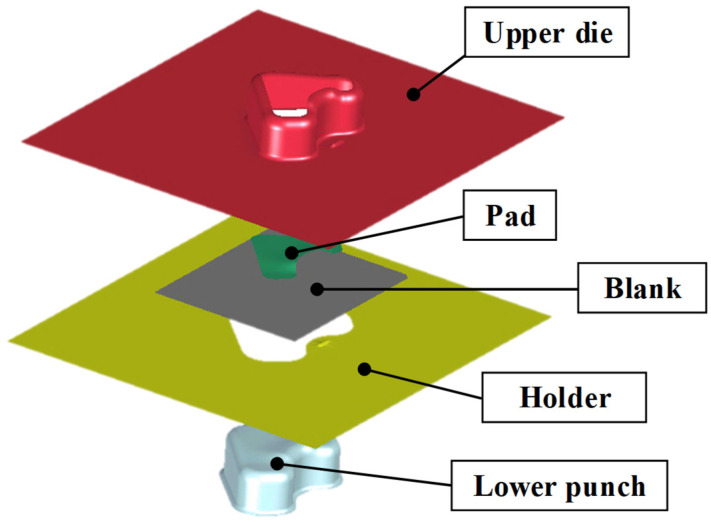
FE model for stamping of VCM sheet.

An FE simulation of the stamping process was conducted to evaluate the formability of the VCM components. Pre- and post-processing were conducted using J-Stamp/NV (JSOL Corporation, Tokyo, Japan), whereas numerical computation was carried out using the LS-DYNA explicit solver (Ansys, South of Pittsburgh, PA, USA). The overall configuration of the FE model is shown in Figure 13. The model geometry was designed to reflect typical product shapes used in household-appliance manufacturing. The simulation model consisted of an upper die, a lower punch, a pad, a holder, and an initial blank. The initial-blank thickness was 0.8 mm, and both the tools and blank were modeled using shell elements. A fine mesh size of 1.0 mm × 1.0 mm was applied to the blank to accurately describe the local strain distribution. The material properties of the VCM sheet were assigned based on the results of uniaxial tensile tests, thereby ensuring an accurate representation of the material behavior. The blank holding force was set to 10 t, considering the actual press-operation conditions. Moreover, a friction coefficient of 0.12 was applied between the tool and sheet surfaces.

In this simulation, the tools were modeled as rigid bodies. While recent studies have demonstrated that press and die deformation can impact the dimensional accuracy of stamped parts [29], the primary focus of this study is on predicting delamination and wrinkling limits rather than micron-level geometric tolerances. Given that the stiffness of the 200 t press frame is significantly higher than that of the VCM sheet, the influence of elastic tool deflection on the macroscopic strain paths and delamination onset is considered negligible. Furthermore, assuming rigid tools significantly reduces the computational cost, which is essential for generating the large-scale dataset required for the DNN-based multi-objective optimization framework.

**Figure 14:**
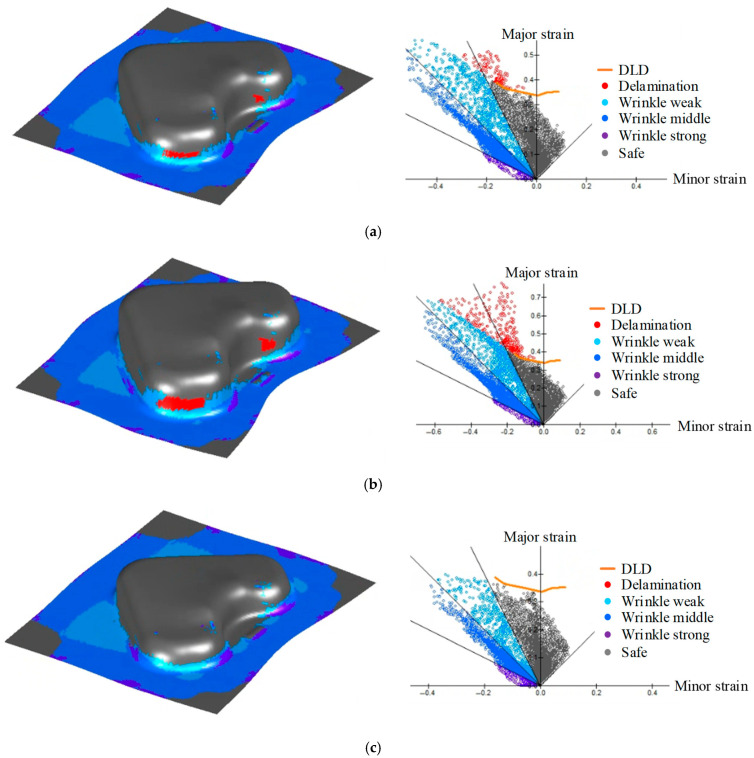
Results of FE simulation for stamping process based on punch stroke. (**a**) 30 mm; (**b**) 25 mm; (**c**) 20 mm.

The stamping die was operated with an adjustable punch stroke to evaluate the formability of the various coated steel grades. The total stroke of the die was 30 mm. However, when the VCM sheet was formed at a full punch stroke of 30 mm, excessive delamination occurred, as shown in Figure 14a, and fracturing was likely to occur. Therefore, an appropriate punch stroke that allowed a formability assessment while preventing severe delamination needed to be determined. To obtain suitable forming conditions, FE simulations were conducted for 10 randomly selected combinations of process variables under different punch-stroke conditions. The results indicated that at a punch stroke of 25 mm, delamination occurred under all the tested conditions, as illustrated in Figure 14b. Conversely, when the punch stroke was reduced to 20 mm, the occurrence of delamination depended on the process parameters. Some conditions exhibited delamination, whereas others remained intact, as shown in Figure 14c. Based on these observations, a punch stroke of 20 mm was selected as the standard forming condition for subsequent simulations and optimization studies of VCM stamping because it provided a balance between sufficient deformation and delamination sensitivity.

### 3.2. Multi-Objective Optimization

A data-driven multi-objective optimization framework was developed by employing a DNN as a surrogate model trained on FE-simulation data. This approach enabled the efficient and continuous exploration of the design space without the need for repetitive simulations. The process variables defined in Section 3.1.1 were used as the design inputs, while the two objective functions, the delamination index (*F_d_*) and wrinkle index (*F_w_*), were simultaneously minimized.

#### 3.2.1. Construction of DNN Surrogate Model

To construct the DNN model, 108 FE simulations were conducted based on a full factorial design of the four process variables and their respective levels. For each simulation, the characteristic values of delamination and wrinkling were evaluated using the safety DLD, and the resulting data were used to establish a training dataset. The constructed dataset was normalized to a range between zero and one to improve learning stability and convergence. The normalized data were randomly divided as follows: 80% for training, 10% for validation, and 10% for testing.

Prior to finalizing the network architecture, a comparative study was conducted using simpler surrogate models, including shallow multilayer perceptrons with fewer layers and neurons. However, these reduced complexity networks consistently produced coefficient of determination (*R*^2^) values below 0.95, particularly in regions exhibiting steep nonlinear changes near the DLD/WLD boundaries. Because the optimization framework requires high-fidelity prediction across the entire design space, an accuracy threshold of *R*^2^ ≥ 0.95 was established. The deep network architecture described below was empirically determined as the minimum configuration required to satisfy this accuracy criterion.

Accordingly, the final DNN model was implemented with five hidden layers, each consisting of 1000 neurons, to adequately learn the complex nonlinear correlations between the proven variables and defect responses. The overall architecture of the developed DNN model is illustrated in Figure 9. It consists of an input layer representing the four process variables, hidden layers, and two output nodes corresponding to the delamination and wrinkling. The ReLU activation function and the Adam optimizer were used for efficient training. The learning rate was set to 0.001, and the maximum number of epochs was set to 300. To mitigate the risk of overfitting inherent in high-capacity models, early stopping was implemented. The predictive performance evaluated using the *MAE*, *RMSE*, and *R^2^*, as summarized in Table 3, confirmed that the trained network reliably captured the nonlinear relationship between the process variables and defect indices without overfitting.

#### 3.2.2. Exploration of Design Space and Pareto Optimization

**Figure 15:**
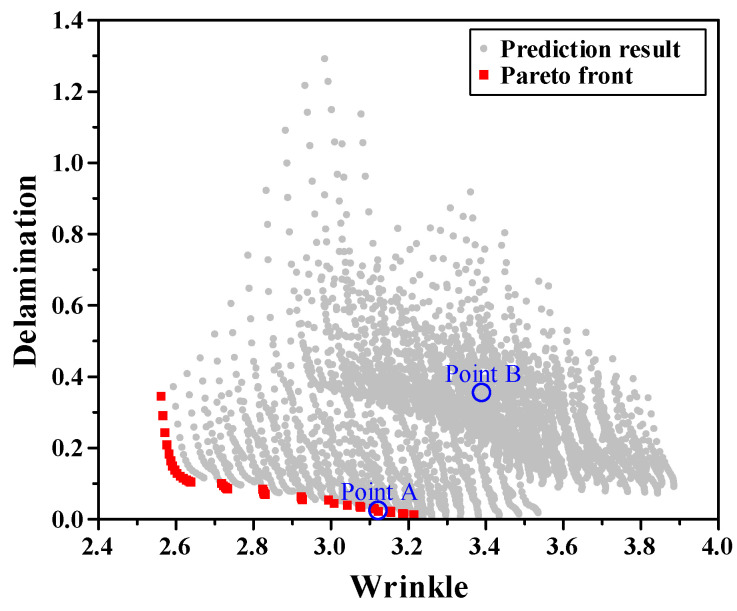
Prediction results and Pareto front for the entire design space.

The design space consisted of four controllable parameters that can be adjusted directly during the stamping process. The blank shape included three geometries, and the initial blank contour was varied from 0 to 10 mm in 1 mm increments to control the amount of material inflow at the sheet edge. The clearance between the punch and die ranged from 0 to 0.15 mm in 0.01 mm steps to account for the contact pressure and flow resistance. The punch radius was set between 4 and 10 mm at intervals of 1 mm to influence the curvature-induced strain localization during deformation. These four variables were combined through a full factorial design, resulting in 3696 physically verifiable design combinations. All combinations were defined within the practical manufacturing limits of the tooling, ensuring that each condition could be experimentally or numerically validated through 1:1 mapping with real forming configurations. Using the trained DNN surrogate model, both delamination and wrinkling were predicted for all 3696 combinations, constructing a complete design space, as illustrated in Figure 15. Each point represents a unique process condition, and the horizontal and vertical axes correspond to *F_w_* and *F_d_*, respectively. Among the 3696 evaluated cases, 44 Pareto-optimal solutions were identified. These solutions collectively formed the Pareto front, as shown in Figure 15. The front revealed a distinct inverse relationship between the two objectives, demonstrating the intrinsic conflict between interfacial adhesion and surface stability in VCM stamping. From the Pareto front, point A was selected as the optimal condition, and one dominated solution, point B, was chosen for comparison. Table 4 summarizes the selected process conditions.

To quantify the computational efficiency of the proposed optimization framework, a runtime benchmark was performed. A single FE simulation required approximately 30 min due to the computational complexity involved in nonlinear contact, draw-bead resistance, and large deformation plasticity. Consequently, evaluating all 3696 combinations using FE alone would require about 1848 h. In contrast, the trained DNN surrogate model predicted the entire design space in less than one minute. This dramatic reduction in computational cost enables rigorous full-space exploration and Pareto extraction, which are otherwise impractical using FE methods alone. Furthermore, this result demonstrates that the proposed approach establishes a scalable optimization framework applicable to more complex forming processes, where a single FE simulation often requires multiple hours.

#### 3.2.3. Validation Using FE Simulation

**Figure 16:**
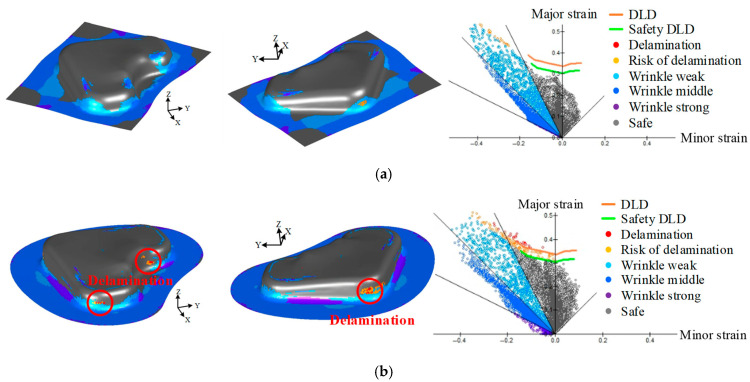
Comparison result of FE simulation for optimal and dominated solutions. (**a**) Optimal condition; (**b**) Dominated condition.

The two selected cases were validated using an FE simulation. The results are presented in Figure 16, which illustrates the strain distribution based on the constructed DLD. As shown in Figure 16a, the optimal solution at point A did not exceed the DLD, confirming that both the delamination and wrinkling were effectively minimized. By contrast, the dominated solution at point B exhibited an excessive strain concentration in the uniaxial tensile mode, resulting in strain values exceeding the DLD, as shown in Figure 16b. These results indicate that the process conditions at point A can be considered the most feasible and optimal forming conditions. Additionally, the characteristic values obtained from the FE simulations were compared with the corresponding values predicted by the trained DNN model. As summarized in Table 5, the comparison revealed a high prediction accuracy, with an error within 4% between the predicted and simulated results. The effectiveness of the proposed DNN–Pareto optimization method was confirmed from the above results. Finally, stamping experiments on the VCM part were performed under the two selected conditions to validate the reliability and practical applicability of the proposed multi-objective optimization framework.

## 4. Experimental Verification

### 4.1. Manufacturing of the VCM Part Through the Stamping Process

**Figure 17:**
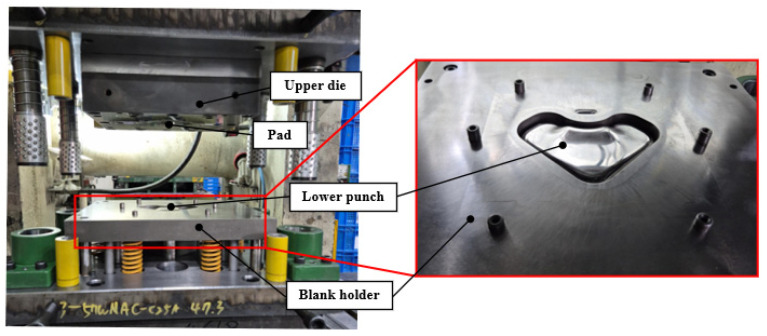
Experimental equipment for the stamping of the VCM sheet.

**Figure 18:**
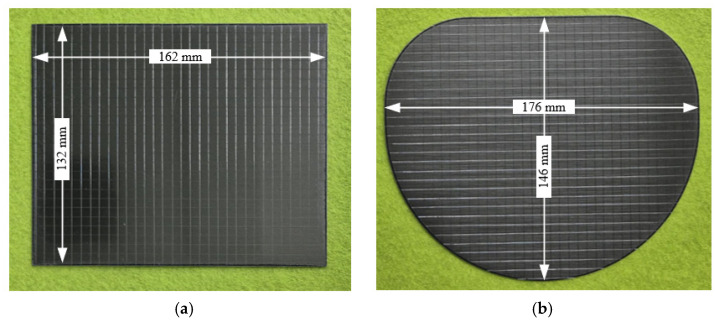
Initial blank shape and applied cross-hatched pattern for stamping process. (**a**) Rectangle; (**b**) Circle.

**Figure 19:**
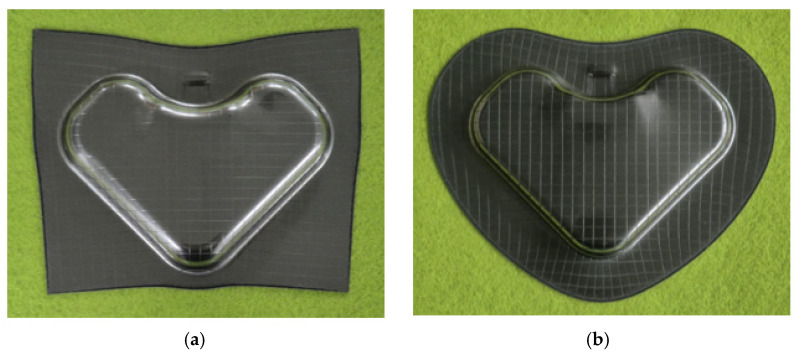
Experimental results of manufacturing through stamping process. (**a**) Rectangle; (**b**) Circle.

In this study, experimental stamping was performed using VCM sheets to validate the effectiveness of the proposed optimization framework. As shown in Figure 17, a 200 t mechanical press was used for the experiments, and the stamping tool consisted of an upper die, lower punch, pad, and blank holder. The forming conditions were the same as those used in the FE simulations with a punch stroke of 20 mm. Two cases of optimal and dominated solutions selected in Section 3.2 were tested. As shown in Figure 18, the VCM sheets were machined into blank shapes corresponding to the selected cases. A cross-hatched pattern was applied to the surface to facilitate the observation of the delamination. The prepared blank was placed on the blank holder and formed using the upper die and lower punch. The VCM parts corresponding to the two cases were formed after stamping, as shown in Figure 19.

### 4.2. Experimental Results of Immersion Test

Immersion tests were conducted on two types of VCM parts manufactured via stamping to verify the effectiveness of the proposed optimization framework. The experimental apparatus used for the immersion tests is shown in Figure 5. Each case was tested at least three times under the same conditions for reliability. Based on the immersion-test standard for VCM sheets, all specimens were immersed in water at 60 °C for 30 min, followed by air drying for 1 h. This test was designed to evaluate the interfacial stability and environmental resistance of stamped VCM parts under high-temperature and high-humidity conditions.

**Figure 20:**
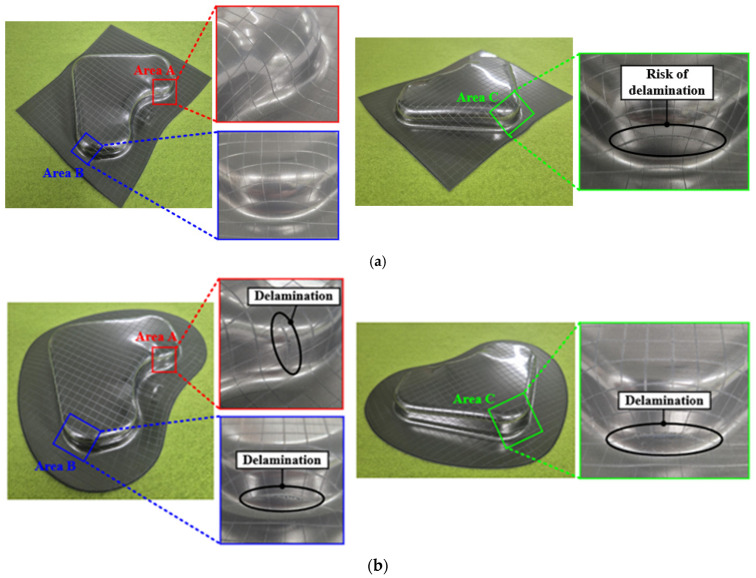
Observation of delamination in each area after immersion test. (**a**) Point A; (**b**) Point B.

Figure 20 shows the results of the immersion tests conducted on the VCM parts. The overall experimental results were in good agreement with the FE simulations. These results confirmed that excessive wrinkling did not occur under either condition. A detailed evaluation of the delamination behavior was performed for three representative areas: A, B, and C. The experimental results for the optimal conditions selected at Point A are shown in Figure 20a. Delamination was not observed in areas A and B, and although the risk of delamination was predicted in area C based on the FE simulation shown in Figure 16a, it did not exceed the DLD. The predictive reliability of the DLD was experimentally validated by confirming the absence of delamination. However, a slight surface distortion was detected near the edge, which was consistent with potential delamination behavior, indicating that delamination may have occurred if the forming depth was further increased beyond the current condition. In contrast, the experimental results for the dominated condition selected at Point B are shown in Figure 20b, and delamination behavior was observed in all three areas. As shown in Figure 16b, delamination was predicted to occur in areas A, B, and C. The experimental results showed a similar trend, with distinct delamination observed in areas B and C. Severe delamination was not observed in Area A. However, slight initiation of delamination was detected near the edge of the cross-hatched pattern. This localized initiation is attributed to the major strain in Area A being very close to the DLD curve. Consequently, only a marginal level of delamination was observed under these conditions.

Overall, the experimental and FE results were consistent, verifying the reliability and validity of the FE simulation. Furthermore, the proposed DNN–Pareto-based multi-objective optimization framework effectively identified the nonlinear relationships between complex process parameters and defect responses. Using this framework, the optimal conditions were successfully derived. The nearly perfect agreement between the predicted and experimental results demonstrated the high predictive accuracy and practical applicability of the proposed model. Therefore, the DLD established in this study can serve as a practical predictive tool for evaluating formability while considering the environmental reliability of VCM parts. In addition, the proposed DNN–Pareto-based multi-objective optimization framework provides an efficient approach for minimizing defects and determining the optimal parameters in VCM forming processes.

While the validation in this study focused on a specific VCM grade and a single component geometry, the proposed DNN–Pareto framework is fundamentally data-driven. Therefore, rather than claiming universal applicability, this work demonstrates a methodological scalability that can be extended to other coated metal systems or multilayer materials systems when valid limit diagrams and FE datasets are constructed. Thus, the core contribution of this study is the establishment of a transferable optimization strategy capable of handling various material constraints.

## 5. Conclusions

This study developed an integrated framework that combines the experimentally established delamination limit diagram (DLD), FE-based defect quantification, and DNN–Pareto multi-objective optimization to determine environmentally reliable stamping conditions for VCM sheets. The main findings are summarized as follows.

First, a DLD was constructed by coupling LDH testing with immersion aging to reflect high-temperature and high-humidity transport conditions. The resulting DLD clearly demonstrated that interfacial delamination occurs at strain levels significantly below fracture, confirming that coating–substrate adhesion governs the true forming limit of VCM sheets. This provides material-specific evidence that environmental degradation must be considered in VCM forming.

Second, FE simulations revealed that delamination initiates from curvature-induced tensile and shear stresses at the coating interface, whereas wrinkles arise from compressive instability driven by excessive material inflow. These mechanisms demonstrated a strong dependency on blank shape, initial contour, tool clearance, and punch radius, highlighting the inherent trade-off between interfacial adhesion and surface stability.

Third, a defect quantification scheme was formulated using safety-corrected DLD and WLD curves, allowing delamination and wrinkling to be expressed as global characteristic indices (*F_d_* and *F_w_*). This enabled consistent evaluation of forming quality across all 3696 process combinations and provided defect-resolved material insights that conventional fracture-based forming limits could not capture.

Fourth, stamping and immersion experiments conducted on optimal and dominated forming conditions verified that the DLD accurately predicted delamination onset under environmental exposure. The optimal condition suppressed both delamination and wrinkling, whereas the dominated condition exhibited multi-region delamination consistent with FE predictions. This confirms that the developed criteria reliably represent real deformation and coating failure behavior.

Finally, the DNN surrogate model accurately reproduced the nonlinear relationships between process parameters and the two defect indices, enabling rapid multi-objective optimization with minimal computational cost. While the DNN serves as a predictive surrogate model rather than a fundamental physical model, its role is to accelerate the evaluation of experimentally grounded defect criteria. Therefore, the primary contributions lie in establishing environmentally conditioned forming limits and integrating them into a computationally efficient optimization framework.

Overall, the proposed DLD–FE–DNN–Pareto framework offers a comprehensive, material-informed methodology for predicting and optimizing forming defects in VCM stamping. The established DLD provides a physically grounded forming-limit criterion under realistic environmental exposure, and the optimized process conditions contribute to improving the reliability, durability, and production quality of VCM-coated sheet components in industrial applications.

## Figures and Tables

**Table 1:** Value of process variables based on levels.

Levels	Process Variables			
	Blank Shape	Initial Blank Contour	Clearance	Punch Radius
1	Rectangle	+0 mm offset	0.00 mm	4 mm
2	Octagon	+5 mm offset	0.05 mm	7 mm
3	Circle	+10 mm offset	0.10 mm	10 mm
4	-	-	0.15 mm	-

**Table 2:** Representative results of objective-function characteristic value results for process variables.

Levels of Process Variables				Objective Functions	
Blank Shape	Initial Blank Contour	Clearance	Punch Radius	Delamination	Wrinkle
1	1	1	3	0.265627	3.073384
1	2	3	2	0.074765	3.067223
1	3	2	1	0.789036	3.014227
1	3	4	3	0.023121	3.537217
2	1	2	3	0.231282	3.395943
2	2	4	2	0.210795	3.409962
2	3	1	3	0.468012	3.782278
3	2	3	3	0.132027	3.639256
3	2	4	3	0.108441	3.652441
3	3	3	1	0.504360	3.414276

**Table 3:** Prediction performance of trained DNN model.

Test Dataset Error		
*MAE*	*RMSE*	*R* ^2^
0.027	0.044	0.954

**Table 4:** Selection results of optimal and dominated solutions.

Point	Process Condition			
	Blank Shape	Initial Blank Contour	Clearance	Punch Radius
A	Rectangle	+1 mm	0.15 mm	9 mm
B	Circle	+8 mm	0.08 mm	5 mm

**Table 5:** Error comparison between FE simulation and predicted value through DNN model.

	Point A		Point B	
	Delamination	Wrinkle	Delamination	Wrinkle
FEM	0.019369	3.011618	0.358579	3.338308
DNN	0.020034	3.121639	0.359897	3.395217
Error (%)	3.5	3.7	0.37	1.71

## Data Availability

The original contributions presented in this study are included in the article. Further inquiries can be directed to the corresponding author.
